# Virologic non‐suppression and early loss to follow up among pregnant and non‐pregnant adolescents aged 15–19 years initiating antiretroviral therapy in South Africa: a retrospective cohort study

**DOI:** 10.1002/jia2.25870

**Published:** 2022-01-15

**Authors:** Patience Nyakato, Michael Schomaker, Geoffrey Fatti, Frank Tanser, Jonathan Euvrard, Nosisa Sipambo, Matthew P. Fox, Andreas D. Haas, Constantin T. Yiannoutsos, Mary‐Ann Davies, Morna Cornell

**Affiliations:** ^1^ Centre for Infectious Disease Epidemiology and Research School of Public Health and Family Medicine University of Cape Town Cape Town South Africa; ^2^ Institute of Public Health Medical Decision Making and Health Technology Assessment UMIT ‐ University for Health Sciences Medical Informatics and Technology Tyrol Austria; ^3^ Kheth'Impilo AIDS‐Free Living Cape Town South Africa; ^4^ Division of Epidemiology and Biostatistics Department of Global Health Faculty of Medicine and Health Sciences Stellenbosch University Cape Town South Africa; ^5^ Africa Centre for Health and Population Studies University of KwaZulu‐Natal Somkhele South Africa; ^6^ Khayelitsha ART Programme and Medecins Sans Frontieres Cape Town South Africa; ^7^ Harriet Shezi Children's Clinic Chris Hani Baragwanath Academic Hospital Soweto South Africa; ^8^ Department of Epidemiology, Boston University School of Public Health Boston University Boston Massachusetts USA; ^9^ Health Economics & Epidemiology Research Office Department of Internal Medicine School of Clinical Medicine Faculty of Health Sciences University of the Witwatersrand Johannesburg South Africa; ^10^ Institute of Social and Preventive Medicine University of Bern Bern Switzerland; ^11^ Department of Biostatistics R.M. Fairbanks School of Public Health Indiana University Indianapolis Indiana USA

**Keywords:** adolescents, antiretroviral therapy, HIV, loss to follow up, pregnancy, virologic non‐suppression

## Abstract

**Introduction:**

Older adolescents aged 15–19 years continue to have high rates of loss to follow up (LTFU), and high rates of virologic non‐suppression (VNS) compared to younger adolescents and adults. Adolescent females are at risk of pregnancy, which puts those living with HIV at a dual vulnerability. Our study assessed the factors associated with VNS and LTFU in older adolescents (including pregnant females) who initiated antiretroviral therapy (ART) in South Africa.

**Methods:**

We included adolescents aged 15–19 years initiating ART between 2004 and 2019, with ≥ one viral load (VL) measurement between 4 and 24.5 months, and ≥ 6 months follow‐up, from six South African cohorts of the International epidemiology Databases to Evaluate AIDS‐Southern Africa (IeDEA‐SA). We defined VNS as VL ≥400 copies/ml and LTFU as not being in care for ≥180 days from ART start and not known as transferred out of the clinic or dead in the first 24 months on ART. We examined factors associated with VNS and LTFU using Fine&Gray competing risk models.

**Results:**

We included a total of 2733 adolescents, 415 (15.2%) males, median (IQR) age at ART start of 18.6 (17.3, 19.4) years. Among females, 585/2318 (25.2%) were pregnant. Over the 24‐month follow‐up, 424 (15.5%) of all adolescents experienced VNS: range (11.1% pregnant females and 20.5% males). Over half of all adolescents were LTFU before any other event could occur. The hazard of VNS reduced with increasing age and CD4 count above 200 cells/μl at ART initiation among all adolescents having adjusted for all measured patient characteristics [adjusted sub‐distribution hazard ratio (aSHR) 19 vs. 15 years: 0.50 (95% CI: 0.36, 0.68), aSHR: >500 vs. ≤200 cells/μl: 0.22 (95% CI: 0.16, 0.31)]. The effect of CD4 count persisted in pregnant females. Increasing age and CD4 count >200 cells/μl were risk factors for LTFU among all adolescents.

**Conclusions:**

Older adolescents had a high risk of LTFU shortly after ART start and a low risk of VNS, especially those initiating treatment during pregnancy. Interventions addressing adherence and retention should be incorporated into adolescent‐friendly services to prevent VNS and LTFU and endeavour to trace lost adolescents as soon as they are identified.

## 1 INTRODUCTION

In 2019, about 1.7 (1.1–2.4) million adolescents aged 10–19 years and 3.4 million youth aged 15–24 years were living with HIV worldwide [1], with the majority living in sub‐Saharan Africa (SSA). In recent years, adolescent‐friendly HIV programs have increased as antiretroviral therapy (ART) programmes become decentralized [2, 3]. Despite these efforts, older adolescents aged 15–19 years continue to have high rates of loss to follow up (LTFU), less than optimal adherence and high rates of virologic non‐suppression (VNS) compared to younger adolescents aged 10–14 years and adults [4, 5, 6].

Behavioural and patient characteristics play an important role in adherence to ART and retention in care among adolescents living with HIV. Adolescence is when sexuality, gender norms and sexual relationships are explored while experiencing major physical and physiological changes [7, 8]. These changes often influence adolescents' health‐related behaviour, including clinic attendance and long‐term adherence to HIV medication, which affects virologic suppression (VS) [9]. In Gauteng, South Africa, older adolescents and young adults (20–24 years) were more likely to have VNS and virologic failure compared to either younger adolescents or adults [10]. Transition to adult care generally happens during later adolescence, and in some settings, older adolescents are often treated as adults [11, 12]. In these situations, they would be expected to make healthcare decisions without understanding the importance of sustained adherence and retention in care. Among South African adolescents who had successfully transferred, older adolescents were more likely to have VNS and CD4 count cells ≤500 cells/μl compared to younger adolescents at 1‐ and 2‐years post transfer [13].

Globally, adolescent girls and young women aged 15–24 years continue to be disproportionately affected by HIV compared with their male counterparts. Adolescent girls are at a higher risk of acquiring HIV compared to adolescent boys [2], with twice the HIV prevalence of boys and young men in the same age range [1]. Girls are also at risk of pregnancy, which puts those living with HIV at a dual vulnerability, dealing with a chronic illness and the physical and emotional changes that occur during pregnancy [14, 15]. VNS among adolescent girls living with HIV is of particular concern due to the high risk of virologic failure, morbidity and mortality, and the potential for HIV transmission to an unborn child should they become pregnant [15]. Studies have reported high rates of LTFU among pregnant and breastfeeding women, which are associated with a high risk of VNS [16, 17]. To date, however, there are limited data on VNS among adolescents (pregnant and non‐pregnant) who initiate ART between the ages of 15 and 19 years in SSA [18, 19]. Our study aimed to assess the factors associated with VNS and LTFU in older adolescents (including pregnant females) who initiated ART between 2004 and 2019 in the South African cohorts of the International epidemiology Databases to Evaluate AIDS‐Southern Africa (IeDEA‐SA) collaboration.

## 2 METHODS

### 2.1 Study population and inclusion criteria

**Figure 1:**
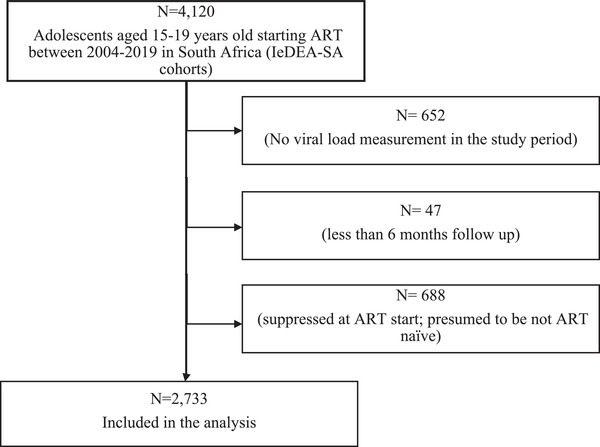
Flow chart showing the inclusion and exclusion criteria of adolescents aged 15–19 years old in International epidemiology Databases to Evaluate AIDS‐South African cohorts.

We included all ART‐naïve (defined as not being on any ART regimen) adolescents aged 15–19 years initiating ART between 2004 and 2019 from six South African cohorts of the IeDEA‐SA collaboration [20, 21]. These cohorts are in both rural (Hlabisa) and urban (Harriet Shezi, Themba Lethu, Khayelitsha, Kheth'Impilo and AfA) locations entailing public hospitals (Harriet Shezi, Themba Lethu), public sector primary healthcare centre (Khayelitsha) not‐for‐profit organizations (Kheth'Impilo, Hlabisa) and one private sector managed‐care HIV program (AfA), across three South African provinces: the individual cohort profiles have been described elsewhere [22, 23, 24, 25, 26, 27]. All facilities have a mix of paediatric and adult populations except for Harriet Shezi, a paediatric hospital. To be included in the analysis, these adolescents had to have at least one viral load (VL) measurement between 4 and 24.5 months on ART, and ≥ 6 months follow‐up on ART (Figure 1). We excluded 688 adolescents with a suppressed VL at ART start (VL<400 copies/ml) as they were unlikely to be ART‐naïve.

### 2.2 Outcome definitions

The main outcome of interest was the first non‐suppressed VL (VNS) within 4–24.5 months from ART start. We defined VNS as any VL ≥400 copies/ml (cpm). The choice of the 400 cpm cut off is based on historical accuracy of the test: VL assays used to only be reliable at around 400 cpm for the years included.

#### 2.2.1 Competing outcomes

We considered LTFU (defined as the first gap in care of ≥180 days from ART start and not known as having transferred out of the clinic or died in the study period with no consideration for patients cycling in and out of care within this period), all‐cause mortality and official transfers. Most of these facilities trace patients who are lost as part of routine procedures. Mortality is defined as all‐cause mortality. We examined these outcomes among all adolescents and separately among pregnant females. Pregnancy is routinely collected and recorded in patient files and electronic databases.

### 2.3 Analysis

We used descriptive statistics of proportions, medians, interquartile ranges (IQRs), rates and cumulative incidence functions to summarize data stratified by sex (males, non‐pregnant females and pregnant females) [28]. We examined factors associated with VNS using Fine&Gray competing risks regression models among all adolescents and separately among pregnant females [29]. We included characteristics recorded at ART initiation [sex, World Health Organization (WHO) Stage, CD4 count, health facility, calendar year of ART start and age at ART start] in the model. We report adjusted sub‐distribution hazard ratios. In our secondary analysis, we examined factors associated with being LTFU in the presence of death and official transfers as competing events among all adolescents and separately among pregnant females. Under Option B, rolled out in South Africa from 1 April 2013 to 31 December 2014, ART was only recommended for pregnant and breastfeeding females. It is relatively uncommon for adolescent mothers to breastfeed for an extended period as most need to return to school. To address this issue, we censored the analysis at 9 months for women who initiated ART during the rollout of Option B in South Africa [30].

We assumed that missing data were missing at random and we multiply imputed (15 times) missing baseline CD4 count and WHO Stage data using multiple imputations (MIs) with a chained equations approach [31]. Results were then combined using Rubin's rules [32]. We report the MI adjusted results among all adolescents and pregnant females, respectively, for both outcomes of VNS and LTFU. Analysis and data management were performed in Stata version 15.1 (Stata Corporation, College Station, TX, USA).

### 2.4 Ethics statement

The data used in this analysis are collected as part of the standard routine procedure at the facilities; patients or caregivers have given consent to the collection of the data. IeDEA‐SA cohorts have also obtained ethical approval to collect and transfer anonymized data through their respective Institutional Review Boards (IRBs). The IeDEA‐SA data centre has approval from the University of Cape Town's IRB (Human Research Ethics Committee) to receive and analyse these anonymized data.

## 3 RESULTS

### 3.1 Patient characteristics

We included a total of 2733 adolescents, 415 (15.2%) males, with a median (IQR) age at ART start of 18.6 (17.3, 19.4) years (Table 1). Among females, 585/2318 (25.2%) were pregnant at ART initiation. While nearly one in five adolescents were in WHO Stages 3&4, the proportion ranged from 4.1% among pregnant females to 31.3% among males. Similarly, among adolescents with recorded CD4 count, 17.3% had CD4 count ≤ 200 cells/μl, with the highest proportion found among males (29.9%) and the lowest in pregnant females (7.9%). Most adolescents had initiated ART between 2013 and 2019. The majority (56.1%) of adolescents were from Kheth'Impilo, ranging from 44.1% males to 63.6% pregnant females. The median (IQR) number of VL measurements per patient was 1 (1,2): range (1,5) for all sex categories.

**Table 1:** Patient characteristics of adolescents 15–19 years old at ART initiation and outcomes (virologic non‐suppression, loss to follow up, mortality and transfers) at 24 months after ART initiation

Patient characteristics	Total,u *n* (%)ª(*N* = 2733)	Male, *n* (%)ª(*N* = 415)	Non‐pregnant females, *n* (%)ª(*N* = 1733)	Pregnant females, *n* (%)ª(*N* = 585)
Age at ART start, years, median (IQR)	18.6 (17.3, 19.4)	17.2 (16.0, 18.8)	18.6 (17.4, 19.4)	18.9 (18.0, 19.5)
Age at ART start, years				
15	272 (10.0)	99 (23.9)	152 (8.8)	21 (3.6)
16	310 (11.3)	87 (21.0)	188 (10.9)	35 (6.0)
17	433 (15.8)	66 (15.9)	280 (16.2)	87 (15.0)
18	696 (25.5)	74 (17.8)	443 (25.6)	179 (30.6)
19	1022 (37.4)	89 (21.5)	670 (38.6)	263 (45.0)
WHO Stage				
Stage 1&2	1850 (67.7)	204 (49.2)	1136 (65.5)	510 (87.2)
Stage 3&4	537 (19.7)	132 (31.3)	383 (22.1)	24 (4.1)
Missing	346 (12.7)	81 (19.5)	214 (12.4)	51 (8.7)
CD4 count (cells/μl) at ART start				
≤200	472 (17.3)	124 (29.9)	284 (16.4)	46 (7.9)
201–350	762 (27.9)	119 (28.7)	431 (24.9)	155 (26.5)
351–500	598 (21.9)	71 (17.1)	393 (22.7)	142 (24.6)
≥500	807 (29.5)	89 (21.5)	568 (32.8)	215 (36.8)
Missing	94 (3.4)	12 (2.9)	57 (3.3)	25 (4.3)
Calendar year of ART start				
2004–2006	96 (3.5)	14 (3.4)	76 (4.4)	6 (1.0)
2007–2009	277 (10.1)	50 (12.1)	201 (11.6)	26 (4.4)
2010–2012	656 (24.0)	124 (29.9)	438 (25.3)	94 (16.0)
2013–2015	801 (29.3)	89 (21.5)	479 (27.6)	233 (39.8)
2016–2019	903 (33.0)	138 (33.3)	539 (31.1)	226 (38.6)
Current age, years, median (IQR)	19.5 (18.2, 20.4)	18.7 (17.5, 20.0)	19.9 (18.7, 20.8)	19.9 (19.1, 20.8)
Health facility				
AfA	135 (4.9)	49 (11.8)	79 (5.1)	7 (1.2)
Harriet Shezi	76 (2.8)	39 (9.4)	37 (2.1)	0 (0.0)
Hlabisa	384 (14.1)	64 (15.4)	235 (13.2)	85 (14.5)
Khayelitsha	496 (18.2)	51 (12.3)	328 (18.5)	117 (20.0)
Kheth'Impilo	1532 (56.1)	183 (44.1)	977 (55.0)	372 (63.6)
Thembalethu	110 (4.0)	29 (7.0)	77 (4.3)	4 (0.7)
Virologic non‐suppression^b^	424 (15.5)	85 (20.5)	274 (15.8)	65 (11.1)
Secondary outcomes				
In care	747 (27.3)	102 (24.6)	317 (18.3)	147 (25.1)
Transferred out	520 (19.0)	104 (25.1)	382 (22.1)	94 (16.1)
Dead	48 (1.8)	10 (2.4)	37 (2.1)	2 (0.3)
Lost	1418 (51.9)	199 (48.0)	997 (57.5)	342 (58.5)

^ª^ Column percentages are reported.

^b^ VLs ≥400copies/ml.

**Figure 2:**
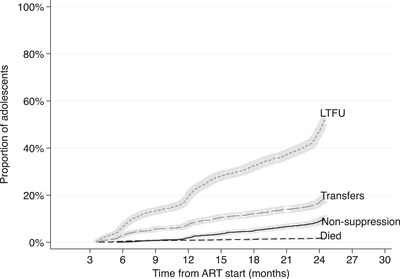
Cumulative incidence functions with 95% confidence intervals of virologic non‐suppression, loss to follow up, transfers and mortality among all adolescents 4–24.5 months from ART start.

**Figure 3:**
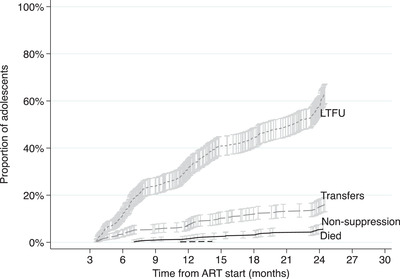
Cumulative incidence functions of virologic non‐suppression, loss to follow up, transfers and mortality among pregnant females 4–24.5 months from ART start.

Over 24‐month follow‐up period, 424 (15.5%) of the adolescents experienced VNS (Table 1, Figures 2 and 3): range, 11.1% among pregnant females to 20.5% among males. At the end of the study period, the estimated mortality was low for all groups (1.8%) and lowest among pregnant females (0.3%). In total, 1418 (51.9%) adolescents were LTFU before any other event could occur, range: 48.0% males to 58.5% pregnant females. Overall, 67.4% of adolescents were either LTFU or had a non‐suppressed VL and 66.5% of pregnant females were either LTFU or had a non‐suppressed VL 24 months from ART start.

The overall estimated rate of VNS among all adolescents was 0.96 [95% confidence interval (CI): 0.88, 1.06] per 100 person‐months (pm) with 44,045 months total time at risk. The estimated rate of VNS among males, non‐pregnant females and pregnant females was: 1.24 (95% CI: 1.00, 1.54), 0.95 (0.84, 1.07) and 0.77 (0.61, 0.99) per 100 pm with 8396 months total time at risk, respectively. The overall estimated rate of LTFU was 3.22 (95% CI: 3.06, 3.39) per 100 pm, highest among pregnant females: 3.86 (3.46, 4.30) per 100 pm and lowest among males: 2.68 (2.32, 3.10) per 100 pm.

### 3.2 Factors associated with VNS and early LTFU within 24 months after ART initiation among all adolescents

The hazard of VNS reduced with increasing age at ART initiation from 16 to 19 years among all adolescents compared to 15 years, having adjusted for all measured patient characteristics [adjusted sub‐distribution hazard ratio (aSHR) 19 vs. 15 years: 0.50 (95% CI: 0.36, 0.68)] (Table 2). The incidence of VNS decreased with increasing CD4 count above 200 cells/μl at ART initiation among all adolescents [aSHR: >500 vs. ≤200 cells/μl: 0.22 (95% CI: 0.16, 0.31)].

**Table 2:** Factors associated with virologic non‐suppression and loss to follow up at 24 months on ART among adolescents who initiate ART at the ages of 15–19 years

	Virologic non‐suppression		Loss to follow up	
Patient characteristics	Adjusted sub‐distribution hazard ratios (aSHRs)^a^	95% confidence interval	Adjusted sub‐distribution hazard ratios (aSHRs)^a^	95% confidence interval
Sex^b^				
Male	1		1	
Female (non‐pregnant)	1.14	0.88, 1.46	0.97	0.82, 1.14
Female (pregnant)	1.21	0.85, 1.72	1.00	0.82, 1.22
Age, years, at ART initiation				
15	1		1	
16	**0.70**	**0.50, 0.99**	1.17	0.92, 1.48
17	**0.57**	**0.40, 0.81**	1.19	0.94, 1.50
18	**0.62**	**0.45, 0.86**	**1.28**	**1.03, 1.59**
19	**0.50**	**0.36, 0.68**	**1.24**	**1.01, 1.53**
WHO Stage at ART initiation				
1&2	1		1	
3&4	1.29	0.98, 1.68	0.91	0.79, 1.06
CD4 count (cells/μl) at ART initiation				
≤200	1		1	
201–350	**0.56**	**0.44, 0.71**	**1.31**	**1.10, 1.56**
351–500	**0.41**	**0.31, 0.55**	**1.43**	**1.19, 1.72**
>500	**0.22**	**0.16, 0.31**	**1.35**	**1.13, 1.62**
Year of ART initiation				
2004–2006	1		1	
2007–2009	0.86	0.54, 1.39	**1.41**	**1.00, 1.99**
2010–2012	1.20	0.77, 1.88	**1.40**	**1.01, 1.95**
2013–2015	1.08	0.69, 1.70	**1.84**	**1.32, 2.56**
2016–2019	1.22	0.74, 1.98	**2.55**	**1.81, 3.57**

^a^ Adjusted for health facility where the patients initiated ART.

^b^ Sex of adolescents refers to the field in the individual patient health record/file.

Bold figures represent effect estimates that were significant at the 5%. level of significance.

After adjusting for other patient characteristics, the hazard of LTFU was higher among adolescents who initiated ART with a CD4 count >200 cells/μl at ART start [aSHR: >500 vs. ≤ 200 cells: 1.24 (95% CI: 1.01, 1.53)], aged 18 or 19 years compared to 15 years and among those who initiated ART between 2013 and 2019 compared to 2004–2006 [aSHR: 2016–2019 vs. 2004–2006: 2.55 (95% CI: 1.81, 3.57)] (Table 2).

### 3.3 Factors associated with VNS and LTFU at 24 months after ART initiation among pregnant females

Pregnant females who initiated ART with a CD4 count above 350 cells/μl had lower hazards of VNS compared to those initiating with a CD4 count ≤200 cells/μl [aSHR: >500 vs. ≤ 200 cells: 0.11 (95% CI: 0.05, 0.27)] having adjusted for all measured patient characteristics (Table 3). Pregnant females initiating ART in WHO Stages 3&4 had a lower hazard of LTFU compared to those initiating in WHO Stages 1&2 [aSHR: 0.52; 95% CI: 0.28, 0.99)]. Figure 3

**Table 3:** Factors associated with virologic non‐suppression and loss to follow up at 24 months among pregnant females who initiate ART at the ages of 15–19 years

	Virologic non‐suppression		Loss to follow up	
Patient characteristics	Adjusted sub‐distribution hazard ratios (aSHRs)^a^	95% confidence interval	Adjusted sub‐distribution hazard ratios (aSHRs)^a^	95% confidence interval
Age, years, at ART initiation				
15–17 years	1		1	
18 years	0.66	0.34, 1.29	1.17	0.86, 1.58
19 years	0.66	0.36, 1.21	0.99	0.74, 1.33
WHO Stage at ART initiation				
1&2	1		1	
3&4	1.25	0.37, 4.28	**0.52**	**0.28, 0.99**
CD4 count (cells/μl) at ART initiation				
≤200	1		1	
201–350	0.62	0.33, 1.18	0.88	0.56, 1.37
351–500	**0.31**	**0.15, 0.65**	1.29	0.83, 2.03
≥500	**0.11**	**0.05, 0.27**	1.43	0.93, 2.20
Year of ART initiation				
2004–2012	1		1	
2013–2015	1.73	0.69, 4.34	0.88	0.62, 1.26
2016–2019	2.07	0.78, 5.43	1.11	0.76, 1.61

^a^ Adjusted for health facility where the patients initiated ART.

Bold figures represent effect estimates that were significant at the 5%. level of significance.

## 4 DISCUSSION

In our study looking at VNS and early LTFU among pregnant and non‐pregnant adolescents initiating ART aged 15–19 years, we found that over 10% of adolescents experienced VNS and more than half were LTFU during the first 24 months from ART start. One in four females had initiated ART during pregnancy. There were differences in characteristics by sex and pregnancy status: males were most likely and pregnant females least likely to experience VNS; while pregnant females were most likely, and males least likely, to be LTFU. We found that initiating ART at older ages and CD4 counts >200 cells/μl were protective against VNS but increased the hazard of LTFU in all adolescents. Among pregnant females, initiating ART at CD4 counts >200 cells/μl reduced the hazard of VNS, and initiating in WHO Stages 3&4 reduced the hazard of LTFU. Adolescents who initiated ART in more recent calendar years were more likely to be LTFU than those initiating ART in 2004–2006.

It is encouraging to see low proportions of VNS in our study although this still falls short of the third UNAIDS 2030 target, that no more than 5% of those on treatment should be virally non‐suppressed [33]. Nonetheless, this proportion was lower than has been reported in other studies, which range from 19% to 73% [34, 35, 36, 37] but concurs with a South African study [36], which found similar rates of VNS for adolescents attending an adolescent‐friendly clinic. VNS is widely used as a proxy for poor adherence [38]. Scott Sutton et al. showed that adherence ≥80% is required for VS for those on single‐tablet regimens and 90% for those on multiple tablet regimens [39]. Our results may, therefore, suggest good adherence, although we were unable to assess this association due to lack of adherence data. Notably, good adherence is associated with low rates of VNS, hence, high rates of VS, which in turn reduces the risk of advanced disease and mortality [40]. Part of the recommended approaches is expansion of adolescent‐friendly services within the health system like peer support, VL monitoring and adherence clubs [41]. MacPherson et al. further identified that adolescent‐friendly services like offering individual and group education and counselling were promising interventions to improve outcomes [42]. We also recommend that future studies collect both adherence and VS data to assess the association between VS and optimal adherence levels in this age group.

The low proportion of VNS among pregnant females is important in low‐ and middle‐income countries, where more than 50% of adolescent births occur [43]. The low rates of VNS translate to a reduced risk of disease progression for the adolescents, and of HIV transmission to both the unborn baby and their sexual partners [44, 45]. Our results concur with a recent population‐based HIV Impact Assessment (PHIA) survey in seven African countries, which reported similar rates of VNS (18%) among adolescent girls and young women living with HIV on ART [46]. However, our results should be interpreted with caution. Due to the high rate of early LTFU in our study, we were unable to observe and measure VS among nearly half of our sample. If we assumed that all pregnant females who were LTFU had VNS, the rates of VNS may have been higher, as reported in the overall rate of VNS (45%) in the PHIA surveys among all adolescent girls and young women regardless of whether they were on ART [47].

Our study confirms that 15‐ to 19‐year‐olds living with HIV are a vulnerable population with high rates of LTFU, especially in the first 24 months after ART initiation [10, 48, 49, 50, 51]. In East Africa, the cumulative incidence of LTFU at 5 years among young adolescents, older adolescents and young adults was 26.6%, 44.1% and 29.3%, respectively [49]. In a South African study, only 29% of adolescents aged 15–19 years were retained in care 24 months after ART initiation [48]. These high rates of LTFU among adolescents are driven by several factors including but not limited to: stigma and discrimination, substance use, school, work and family responsibilities, non‐disclosure of HIV, drug toxicity and high costs of transport to the facility, compounding adolescent concerns about body image, peer pressure, first sexual experience, mental health concerns and developmental changes [8, 50, 52]. Adult clinics do not offer services that are as adolescent‐friendly as paediatric clinics and adolescents may not be ready to be responsible for their own health needs [53, 54, 55, 56]. We, therefore, recommend targeted support for adolescents and integration of adolescent‐friendly services like mental health services, adolescent sexual and reproductive health across the health system.

The high rate of LTFU among pregnant females is of particular concern given that a quarter of the female adolescents in our study were pregnant, and over half were lost to care within 24 months. Pregnancy in an adolescent with HIV may be a sign of systemic or societal failure: a failure to prevent both pregnancy and HIV, even with available prevention measures [43]. Similar results have been found across different settings among pregnant women on life‐long treatment (Option B+) [49, 57]. In a multi‐site analysis, adolescents who were pregnant at ART initiation had almost three times higher risk of LTFU than adults or young adolescents [49]. Within services to prevent mother‐to‐child transmission of HIV in Cape Town, women initiating ART during pregnancy had more than 50% higher hazard of being LTFU compared to those who were already on ART [57]. Some of the observed LTFU is likely due to silent transfers. For example, in Malawi, a study tracing women LTFU on Option B+ found that many patients had actually self‐transferred to other clinics [17]. Similarly, in sample‐based tracing of patients LTFU, a substantial number had transferred to other clinics [32, 58]. Pregnant adolescent girls who are newly diagnosed with HIV represent a vulnerable population urgently requiring linkage into targeted care, to prevent early LTFU. Paediatric ART programs should also put in place integrated services that cater to the unique needs of pregnant adolescent girls like Prevention of mother‐to‐child transmission (PMTCT).

In our study, older age at ART start was protective against VNS for all adolescents. In contrast to our findings, other studies have documented an increased risk of VNS with increasing age [38, 39, 50, 59]. This difference may be due to differences in comparison groups: most other studies compared older adolescents (15–19 years) with younger adolescents (10–14 years), young adults (20–24 years) and adults, but did not include 15‐ to 19‐year‐olds as a group [13, 60]. It is likely that as adolescents grow older, they become more responsible, more adherent to medication and consequently less likely to experience VNS. This is reassuring especially as adolescents transition to adult care where there is less parental or healthcare worker control.

Adolescents who were healthier at ART initiation, with higher CD4 counts, were less likely to experience VNS but more likely to experience LTFU than peers with more advanced HIV disease. Our finding makes intuitive sense as adolescents with high CD4 counts may feel healthy and unmotivated to attend clinic visits and may become lost to care. Because LTFU is associated with an increased risk of poor adherence, VNS, disease progression and mortality [4, 10], adolescent‐friendly clinics and healthcare systems should develop innovative ways of targeting adolescents aged 15–19 years, particularly those starting in pregnancy, and those with more advanced HIV disease. Such strategies could include differentiated service delivery for both pregnant and non‐pregnant females, adherence clubs, peer support for pregnant adolescent girls as part of the peer mentorship program, and continued counselling to improve treatment outcomes [61]. With the introduction of universal Test and Treat [62], many countries, including South Africa, no longer measure CD4 count at ART start since there is no eligibility requirement for ART initiation. However, given that CD4 count predicted both VNS and LTFU in adolescents 15–19 years old, CD4 count at ART start should still be used to identify vulnerable patients in this age group and provide them with additional support to be retained in care and adherent to medication.

Finally, the increased risk of LTFU among adolescents enrolled in more recent years could be due to crowded health systems or silent transfers due to decentralization of the health system [49, 63]. HIV care services should, therefore, provide additional support to ensure that adolescents starting treatment in more recent years remain in care [64].

To the best of our knowledge, this is the first study looking at HIV treatment outcomes among 15‐ to 19‐year‐old adolescents, and within pregnant females in this age group. Our study is strengthened by the large sample size, wide geographical coverage and the inclusion of public and private located in both rural and urban public health facilities in South Africa, making the results generalizable to similar resource‐limited routine care settings with VL monitoring in SSA. Our study's major limitation was the lack of data on adherence, preventing us from assessing an association between adherence and VNS. We addressed the issue of missing data, a common challenge in observational studies, using MIs. Our results may be subject to selection bias, as adolescents who stayed in care long enough to have VLs taken were already adherent to treatment, hence, the low rates of VNS observed. We did not undertake time‐updated analysis and could not assess incident pregnancies and re‐engagement in care for those who may have returned after the first gap in care. Results should be interpreted cautiously due to the potential for outcome misclassification of deaths and “silent” transfers as LTFU. Adolescents considered lost may have died or are continuing care elsewhere [58]. Therefore, ignoring outcomes among those who are LTFU may lead to under ascertainment of both mortality and retention estimates; two of the main indicators of program effectiveness. A further limitation is that we did not have data on transmission mode and were unable to differentiate between adolescents with perinatally acquired HIV (who would probably be slow progressors) [65, 66] and adolescents with non‐perinatally infected, who may have different barriers to retention. We could not assess if the high rate of LTFU among pregnant females was pre or postpartum.

## 5 CONCLUSIONS

Our study showed that older adolescents initiating ART aged 15–19 years had a high risk of LTFU shortly after ART start and a low hazard of VNS, especially those initiating treatment during pregnancy. Given the heterogeneity in treatment outcomes across these age bands, age‐disaggregated outcomes among adolescents (10–14 vs. 15–19) and young adults (20–24) should be reported. Interventions addressing adherence and retention should be incorporated into adolescent‐friendly services to prevent VNS and LTFU and endeavour to trace lost adolescents as soon as they are identified. Paediatric ART programs should also put in place integrated services that cater to the unique needs of pregnant adolescent girls to prevent mother‐to‐child transmission and offer reproductive health services.

## COMPETING INTERESTS

All authors have no competing of interests.

## AUTHORS’ CONTRIBUTIONS

PN, MS and MD conceptualized the study idea. PN performed the data analysis with guidance and contributions from MS. PN, MC and MAD drafted the article with revisions and comments from all authors. GF, FT, JE, NS, MPF and ADH collected the data. All authors have read and approved the final article.

## FUNDING

Research reported in this article was supported by the Eunice Kennedy Shriver National Institute of Child Health and Human Development (NICHD), the National Institute of Allergy and Infectious Diseases (NIAID) under award number: U01AI069924 and the Center for Infectious Diseases Research Institute (CIDRI)‐Africa Wellcome Centre.

## DISCLAIMER

The content is solely the responsibility of the authors and does not necessarily represent the official views of the National Institutes of Health.

## Data Availability

Data used in this manuscript is available through formal data request form developed by IeDEA global Data harmonisation team working group and can be obtained from www.iedea‐sa.org.
